# Exploring the Laws of Developmental Direction Using a Documented Skeletal Collection

**DOI:** 10.1002/ajpa.25047

**Published:** 2024-12-25

**Authors:** Jennifer S. Nelson, Lesley Harrington, Emily Holland, Hugo F. V. Cardoso

**Affiliations:** ^1^ University of Alberta Edmonton Alberta Canada; ^2^ Brandon University Brandon Manitoba Canada; ^3^ Simon Fraser University Burnaby British Columbia Canada

**Keywords:** children, development, developmental plasticity, growth, stunting

## Abstract

**Objectives:**

Many human growth studies note a trend of differential variation in limb segment lengths, where distal elements show greater variability than their proximal counterparts. This has been attributed to their developmental sequence, where bones further from the head develop later and are more impacted by fluctuating growth conditions. We aimed to explore limb dimensions within this framework, known as the laws of developmental direction, in children (0.09–11.75 years) from a documented skeletal collection of low socioeconomic status.

**Materials and Methods:**

*Z*‐scores were generated for diaphyseal length measurements of six limb bones. Differences between mean *z*‐score values of the limbs, as well as of the proximal and distal segments of each limb, were assessed using paired samples *t*‐tests.

**Results:**

The lower limb was significantly more stunted in growth relative to the upper limb (*p* ≤ 0.001), as was the distal segment of the upper limb relative to the proximal segment (*p* ≤ 0.001). In contrast, the distal segment of the lower limb was significantly less stunted in growth relative to the proximal segment (*p* ≤ 0.001).

**Discussion:**

The findings of increased sensitivity in the lower limb relative to the upper limb and in the distal segment of the upper limb relative to its proximal segment are consistent with the laws of developmental direction. However, the finding of greater sensitivity in the proximal segment of the lower limb relative to the distal segment does not align with the theorized developmental gradient. These results reveal the complexity of human growth and developmental plasticity in response to biocultural factors.

## 1 Introduction

Organisms respond to environmental stress through developmental plasticity, where the growth of critical organs are prioritized over the development of less vital tissues (Barker 2012; Bogin and Varela‐Silva 2010; Temple 2014). This adaptive response has been suggested as the mechanism behind diminished skeletal growth when an individual experiences deleterious developmental conditions, such as malnutrition and disease. As a result of this plasticity, the human skeleton provides a record of exposures to biocultural stressors, enabling reconstructions of the biocultural environment of individuals from their physical remains.

Many studies (Bogin et al. 2002; Holliday 1999; Higgins and Ruff 2011; Jantz and Jantz 1999; Meadows and Jantz 1995; Tanner 1978) which have examined skeletal growth in both living and past populations have observed a trend of differential variation in the length of limb segments, where the distal segment of both the upper and lower limbs tends to be more variable relative to the proximal segment. In particular, the length of the tibia is frequently found to be the most variable of all long bones (Auerbach and Sylvester 2011; Bogin et al. 2002; Meadows and Jantz 1995; Schweich 2000; Ulijaszek 1998). This has been interpreted as evidence that the tibia is more sensitive to biocultural stressors than other long bones (Cardoso and Magalhães 2011; Gowland 2015; Jantz and Jantz 1999). It has also been suggested that this increased sensitivity extends to the entirety of the lower limb, with studies of modern samples demonstrating greater variability in the length of the lower limb relative to that of the upper limb (Bogin et al. 2002; Bogin and Varela‐Silva 2010; Buschang 1982; Frisancho 2007; Li, Dangour, and Power 2007; Jantz and Jantz 1999; Tanner et al. 1982). Some studies prioritize the use of tibial measurements over other long bones, based on the rationale that these data have the highest potential for detecting growth disruption (DeWitte 2018; Lampl, Kuzawa, and Jeanty 2003; Mensforth 1985; Newman, Gowland, and Caffell 2019; Pomeroy et al. 2014).

Although the reasons behind this differential sensitivity remain unclear, there have been several explanations proposed, including reduced blood flow (Pomeroy et al. 2012; Holliday and Ruff 2001; Bogin and Varela‐Silva 2010), thermal regulation (Holliday and Ruff 2001; Temple 2008; Pomeroy et al. 2012), and the cephalocaudal principle of growth (Bogin and Varela‐Silva 2010; Schweich 2000; Jantz and Owsley 1984). This latter hypothesis is frequently cited in studies focusing on childhood health, where the observed developmental variability is attributed to the differing tempos of growth in different parts of the body (Seeman 1997; Smith and Buschang 2005; Bogin and Varela‐Silva 2010). Along with all other mammals, the growth of the human body follows a cephalocaudal sequence, where those tissues located at the superior or cranial end develop in advance of those located toward the “tail” (Kingsbury 1924). A similar gradient is observed in the growth of the appendicular skeleton, which develops in a proximodistal sequence (Scammon 1923). Together, these two developmental trends have been termed the laws of developmental direction (Kingsbury 1924; Scammon 1923). As a result, those portions of the body located nearest to the head and trunk are farther along in their development at birth in relation to final adult size (Humphrey 1998). Elements located farthest from the head and trunk are less developed at birth, and experience more rapid postnatal growth as a result (Kingsbury 1924). It is widely accepted that those skeletal elements undergoing the fastest rate of growth are more sensitive to environmental perturbations than slower growing elements (Cardoso and Magalhães 2011; Bogin 1999; Eveleth and Tanner 1990; Smith and Buschang 2004). As the bones of the lower limb undergo growth more rapidly than those in the upper limb (Buschang 1982; Cameron, Tanner, and Whitehouse 1982; Jungers, Cole, and Owsley 1988; Smith and Buschang 2005; Bogin and Varela‐Silva 2003), they are more affected by deleterious growth conditions during infancy and childhood. This is also true of the distal limb segments, which experience a faster rate of growth relative to the proximal limb segments (Schweich 2000; Buschang 1982; Cameron, Tanner, and Whitehouse 1982; Smith and Buschang 2005).

This study explores relative limb segment length within the theoretical framework of the laws of developmental direction, using a documented skeletal collection of known socioeconomic status (SES). Dating to the turn of the 20th century, the Certosa collection consists of individuals known to be from the less advantaged social classes of Bologna, Italy (Belcastro et al. 2017). In addition to their impoverished background, the collection is well‐suited for research of this nature as the chronological age and sex of each individual is documented from associated birth and death records, permitting precise comparisons of attained skeletal size for age with available reference standards. These comparisons facilitate the detection of discrepancies between the diaphyseal length of sample individuals relative to the mean length‐for‐age from a reference standard. In cross‐sectional studies of growth, such as the present study, these discrepancies in attained length‐for‐age are interpreted as evidence of skeletal growth, with negative discrepancies considered as indicators of growth disturbances.

If those bones located farthest from the trunk are more sensitive to those biocultural insults which affect growth, it is expected to see greater growth delays in the lengths of the bones of the distal limb segment (the tibia/fibula and radius/ulna) relative to the proximal limb segment (the femur and humerus). Given the faster growth of the lower limb versus the upper limb, it is also expected that there will be greater growth delays in the lengths of the bones of the lower limb relative to those of the upper limb. The goals of this study are, first, to examine evidence of differential growth in limb lengths to test if greater relative sensitivity to poor growth conditions is observed in the lower limb within the study sample. This is evaluated through comparisons of diaphyseal length of the bones of the lower limb (the femur, tibia, and fibula) relative to those of the upper limb (the humerus, radius, and ulna). Second, growth stunting in the distal limb segments is evaluated in relation to the proximal limb segments.

By evaluating the growth of these individuals in the context of the laws of developmental direction, it is intended that a greater understanding of how various environmental stressors, such as disease and inadequate nutrition, may be responded to through developmental plasticity and the potential impact this might have on growth of the limb segments. These interpretations of the biocultural growth environment will be strengthened by the age and sex comparisons made possible through the archival documentation of each child from this stratum of late 19th century Bolognese society. It is anticipated that through this research, an increased understanding of how to accurately interpret information pertaining to an individual's health and biocultural environment from their skeletal remains will be achieved.

## 2 Materials and Methods

The study sample was selected from the identified skeletal assemblage of known age and sex individuals amassed from the Certosa Cemetery in Bologna, a large city located in the north of Italy. The collection was created for research and teaching purposes in the mid 1900s and comprises 425 individuals who lived and died in the late 19th to early 20th centuries (Belcastro et al. 2017). These individuals range in age from birth to 91 years of age at the time of their death, with approximately equal numbers of females (45%) and males (55%) represented. Based on their inhumation in those areas of the Certosa Cemetery reserved for impoverished individuals (Vidor 2012), the collection is taken as representing Bologna's poorest social class. Archival research of civil and church records, housed at the Archivio di Stato di Bologna and Archivio Generale Arcivescovile di Bologna, respectively, conducted by the authors in the past several years has supported the assessment of these individuals being from an impoverished socioeconomic background. These documents included both birth and baptismal documents, where many of the adult males in the collection were recorded as being employed as unskilled laborers, while the occupation for the majority of females is listed as housewife (Belcastro et al. 2017).

This study examines those individuals in the collection documented as under the age of 12 years according to their death record. The age and sex of the Certosa children are determined from archival documents, including birth, death, and baptism records. These documents permitted the calculation of chronological age at death to the nearest day for all but five individuals, whose age at death could be determined only to the nearest year. The decision to include only those children younger than 12 years of age was made to ensure comparability between the study sample and the long bone reference length data by including only children with unfused long bone epiphyses. Individuals where damage or breakage may have impacted accurate measurement of diaphyseal length were excluded from the study, as were individuals with obvious skeletal pathological conditions, such as rickets. Forty‐six individuals from the Certosa collection were included in this study. Females and males were evaluated using sex‐specific reference data according to their sex as listed on associated archival records. Chronological age was analyzed in years by conversion to decimal age from birth and death dates. Table 1 provides the age and sex composition of the study sample.

**TABLE 1:** Age and sex composition of the study sample.

Age (years)	Total	Female	Male
0	3	2	1
0.5	9	7	2
1	13	8	5
2	4	2	2
3	4	1	3
4	1	1	0
5	5	4	1
6	1	1	0
7	2	1	1
8	0	0	0
9	2	1	1
10	0	0	0
11	2	0	2
Total	46	28	18

The maximum diaphyseal lengths of the humerus, radius, ulna, femur, tibia, and fibula were measured to the nearest tenth of a millimeter using digital sliding calipers, or to the nearest millimeter with a standard osteometric board, following accepted protocols (Buikstra and Ubelaker 1994). The left side was used when possible, with the right accepted as a substitute in instances of damaged or missing bones. These instances are noted in the appropriate tables, with the number of right‐side substitutions indicated in the sample size. Two‐sample *t‐*tests were used to assess for differences between results obtained from the left side compared with those from the right side. Summary statistics for diaphyseal length were calculated for all long bones using 1‐year intervals for individuals over 1 year of age, while those under this age were calculated using half‐year intervals. These summary statistics are presented with the sexes pooled in order to provide comparable data with archeological studies of childhood skeletal growth (Mays 1999; Ruff, Carfalo, and Holmes 2013), where biological sex is often unknown. As most body size differences between sexes are due to changes linked to puberty, a prepubertal sample such as the one in this study is not likely to be significantly altered in the reported summary statistics, but rather benefits from the bolstered sample size. Sex‐specific summary statistics for females and males were also calculated and are available in the Supporting Information.

Diaphyseal length data from the study sample were compared to sex‐ and age‐specific reference data drawn from the Denver Child Research Council Study, alternatively known as the Maresh dataset (Maresh 1943, 1955, 1970; Ruff 2003; Schillaci, Sachdev, and Bhargava 2012). The original Maresh sample comprises 175 children living in the Denver area in the mid‐1950s, all of whom were of European ancestry and primarily of a middle‐ to upper middle‐class background (Maresh 1943, 1955). Individuals were examined and radiographed at regular intervals during development, resulting in a database of nearly 700 radiographs documenting diaphyseal growth between 2 months and 12 years of age. These data are widely accepted as being representative of a normal human growth pattern and are frequently used for comparative analyses of long bone growth in archeological contexts (Schillaci, Sachdev, and Bhargava 2012). Recently, these data were updated by Spake and Cardoso (2021), who corrected the measurements for radiographic magnification and adapted the values using polynomial regression to calculate smaller age cohorts of 1 month, permitting more refined analyses. Spake and Cardoso's (2021) values were used as a comparative reference for diaphyseal length in the present study.

Measurement error in diaphyseal length was assessed through observer error tests, where a sample of 20 bones was measured by one observer, and then remeasured by the first observer and a second observer after a period of 2 weeks. The relative technical error of measurement (%TEM) and coefficient of reliability (*R*) were calculated following the method established by Ulijaszek and Kerr (1999). A single calculation of %TEM and *R* was used to compare observations and assess intra‐ and interobserver error. While there is no %TEM level specified as an acceptable standard, an *R* value of > 0.95 is considered to be the acceptable standard for this measure (Ulijaszek and Kerr 1999).

Differences in limb length‐for‐age between the study sample and reference data were assessed through *z*‐score calculations. Comparisons between the study sample and the reference population were done using 1‐month intervals, using sex‐specific age values for the reference sample (Spake and Cardoso 2021). Individuals were assigned to the last attained age threshold. *Z*‐score analysis was employed to standardize the differences in the diaphyseal lengths of the Certosa children and the age‐specific means from the reference data. *Z*‐scores are commonly used in both clinical and biological studies of living children to assess growth, as they provide a quantifiable method of comparing differences within and between groups (WHO 1995). A *z*‐score provides a measure of how many standard deviations (SD) the value from an individual deviates from the expected mean value of the age‐ and sex‐specific reference standard, calculated as *z* = (*X* − *μ*)/*σ* where *X* is the diaphyseal length measurement of the study individual, *μ* is the mean diaphyseal length from the reference sample, and *σ* is the standard deviation of the diaphyseal length from the reference population. For studies assessing growth in children, a *z*‐score of −2 or lower is defined as stunted (WHO 1995). *Z*‐scores were calculated for each long bone diaphysis. The *z*‐score values for the lower limb were calculated as an average of the proximal and distal values [(femur + (tibia + fibula)/2)/2], while upper limb was calculated as an average of the proximal and distal values [(humerus + (ulna + radius)/2)/2]. In addition, a composite *z*‐score statistic (CZS) was calculated as the mean of all diaphyseal length *z*‐scores for each individual. Individuals with *z*‐scores ≤ 5 were considered to be potential outliers and explored further.

Differences between the diaphyseal lengths of the study sample and reference population were assessed through the calculation of long bone length‐for‐age *z*‐scores, which provide a measure of growth faltering controlled for age and sex. Paired samples *t*‐tests were used to assess the significance of differences between the mean *z*‐score values of the upper and lower limbs, as well as between the proximal and distal segments of each limb. The samples for these tests include only individuals with measurable elements from both segments being compared. All tests were done with an alpha level of 0.05. The decision to use the *t*‐test was based on the results of the Shapiro–Wilk test of normality and Breusch–Pagan test of heteroscedasticity. Scatterplots of upper limb length against lower limb length, as well as distal segment length against proximal segment length for both limbs were also created to assess similarities between the study sample and the reference population regarding their limb proportionality.

All individual *z*‐scores were calculated using sex‐specific reference values (Spake and Cardoso 2021), with females and males analyzed separately. The ability to evaluate differences between the sexes is rare in archeological research, where the majority of samples do not have associated records indicating the sex of the individuals. As there are presently no reliable osteological methods for estimating the biological sex of subadult skeletal remains, the present study is afforded a unique opportunity to consider varying biocultural factors experienced by female and male children during their early development, including gendered cultural practices and female physiological buffering. *Z*‐score results between the females and males of the collection were assessed for significance of difference using an independent samples *t*‐test. All statistical procedures were performed using the statistical packages jmv and moretests in jamovi (version 2.2; The jamovi project 2021).

To determine if differences in diaphyseal long bone lengths between the study sample and reference data, as well as patterns of growth faltering, are attributable to the appropriateness of the Maresh dataset as a reference standard, comparative analyses were conducted using age prediction formulae from an alternative dataset, which is derived from a pooled‐sample of children within the Lisbon, Spitalfields, and St. Bride's Church skeletal collections (Cardoso, Abrantes, and Humphrey 2014). No datasets could be identified which provide mean length measurements for all six unfused long bones, therefore it was not possible to calculate all necessary *z*‐scores as a method of comparison. All individuals within the alternative dataset were of European descent and are considered to be from socioculturally comparable contexts. These formulae were developed for the purpose of estimating age based on long bone development for a wide range of populations, but are particularly applicable for populations of a similar time period and region, such as the children of the Certosa collection. As the long bone lengths from the study sample fall within the range suitable for the age estimation formulae, they were considered to serve as an appropriate standard of comparison. Using sex‐specific age prediction formulae developed by Cardoso, Abrantes, and Humphrey (2014), the expected diaphyseal length based on known age was calculated by reversing the terms of the classical calibration equations for the estimation of age. The sign and magnitude of discrepancies between actual length and predicted length were compared and used to detect diminished growth in the bones of the sample individuals. These results were then compared to those produced using the Maresh dataset to assess for consistency in the study findings.

## 3 Results

### 3.1 Tests of Measurement Error

Results of the intra‐observer and interobserver error tests indicate that less than 1% of variation in the sample data is attributable to observer error (Table 2).

**TABLE 2:** Intra‐ and interobserver error test results for diaphyseal length measurements.

Intra‐observer		Interobserver	
%TEM	*R*	%TEM	*R*
2.16	0.998	2.08	0.998

Abbreviations: %TEM, relative technical error of measurement; *R*, coefficient of reliability.

### 3.2 Skeletal Growth Analysis

Summary statistics for diaphyseal length are included in Table 3. Data for each long bone are presented separately, with the sexes pooled and the sample broken down into 1 year age intervals (e.g., 1 = 1.0–1.9 years of age), save for those individuals under the age of 1 year, who are presented in half year intervals. Sex‐specific summary statistics are provided in Supporting Informations S1 and S2.

**TABLE 3:** Summary statistics of diaphyseal length (mm) for each long bone (sexes pooled).

Age (years)	Humerus			Radius			Ulna		
	*n*	$\bar{x}$	SD	*n*	$\bar{x}$	SD	*n*	$\bar{x}$	SD
0	3 (1)	70.35	2.84	3 (1)	53.65	1.79	2	60.44	0.85
0.5	7 (3)	83.70	8.57	7 (3)	62.76	4.88	5 (3)	73.85	3.62
1	12 (3)	107.08	11.44	8 (2)	77.57	11.15	7	87.44	11.07
2	4 (1)	117.56	9.57	3	87.84	8.62	3 (1)	96.76	11.03
3	3	136.75	3.04	1	101.54	/	2	108.31	0.40
4	1	156.44	/	1	109.57	/	1	121.22	/
5	4	155.77	11.68	4	112.96	10.45	4	123.19	11.86
6	1	171.77	/	1	122.2	/	1	131.45	/
7	2	190.57	3.44	2	137.97	5.01	2	149.35	5.17
8	/	/	/	/	/	/	/	/	/
9	2	191.41	15.68	2 (1)	140.86	16.36	2	152.91	13.35
10	/	/	/	/	/	/	/	/	/
11	2 (1)	214.29	9.49	2	150.59	6.94	2	166.02	10.58

Age (years)	Femur			Tibia			Fibula		
	*n*	$\bar{x}$	SD	*n*	$\bar{x}$	SD	*n*	$\bar{x}$	SD
0	3 (1)	81.36	4.63	3 (1)	68.77	4.14	3	65.70	4.53
0.5	9 (1)	107.11	13.55	8 (2)	89.88	11.17	6	87.61	9.46
1	13 (4)	132.30	13.90	13 (2)	109.18	12.28	9 (3)	106.38	15.14
2	4	150.32	16.26	4	121.96	14.66	4	117.11	12.99
3	4	172.78	3.23	3	143.63	4.07	2	138.2	2.23
4	1	206.50	/	1	206.50	/	1	164.47	/
5	5 (1)	216.40	11.25	5 (1)	174.28	10.25	5	170.73	9.60
6	1	246.00	/	1	191.43	/	1	187.71	/
7	2	263.25	15.20	2 (1)	210.75	11.67	2	211.25	13.08
8	/	/	/	/	/	/	/	/	/
9	2	255.00	19.80	2	210.75	20.86	2	208.50	22.63
10	/	/	/	/	/	/	/	/	/
11	2	283.50	2.83	2	233.25	12.37	2	230.00	9.19

*Note:* Values for sex‐specific summary statistics are available in Supporting Informations S1 and S2.

Abbreviations: *n*, number of individuals (the number of individuals where the right side was substituted for the left is indicated in parentheses); SD, standard deviation for diaphyseal length (mm); $\bar{x}$, mean diaphyseal length (mm).

**FIGURE 1:**
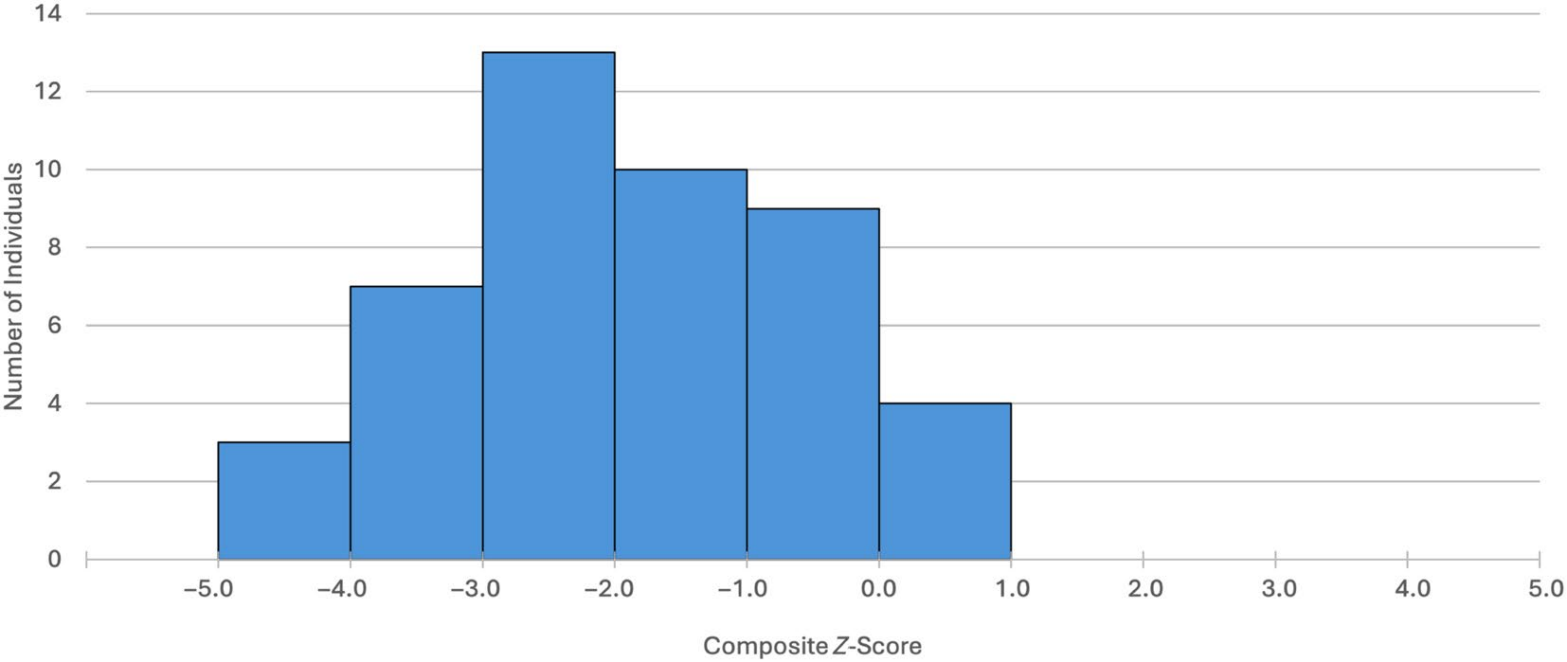
Distribution of composite *z*‐score values with the sexes pooled (*n* = 46).

The results of the *z*‐score summary statistics for the sample presented separately by bone (Table 4) show a difference in diaphyseal lengths between the study sample and the reference population, with the Certosa children exhibiting a delay in skeletal growth relative to the updated Maresh data (Spake and Cardoso 2021). Diaphyseal lengths from the sample individuals show a substantial growth deficit relative to the reference population with a mean *z*‐score = −2.00 which is the globally recognized value for stunted growth (WHO 1995). The children in the sample population are smaller than the reference individuals in all diaphyseal lengths, with 50% (*n* = 23) of individuals producing a CZS value ≤ −2. Analysis of the sample distribution for CZS values demonstrates that very few children in the study sample have positive values (Figure 1). The difference between CZS values and zero was shown to be significant using a one sample *t*‐test (*p* = < 0.001).

**TABLE 4:** Long bone *z*‐scores summary statistics (sexes pooled).

Bone	*n*	$\bar{x}$	Median	SD
Humerus	41	−1.47	−1.50	1.3022
Radius	34	−2.14	−1.93	1.3137
Ulna	33	−2.43	−2.37	1.3133
Femur	46	−2.50	−2.47	1.4310
Tibia	44	−1.86	−1.99	1.3938
Fibula	37	−2.10	−2.22	1.4238
CZS	46	−2.01	−1.99	1.3120

Abbreviations: *n*, number of individuals; SD, standard deviation for *z*‐score values; $\bar{x}$, mean *z*‐score value.

**FIGURE 2:**
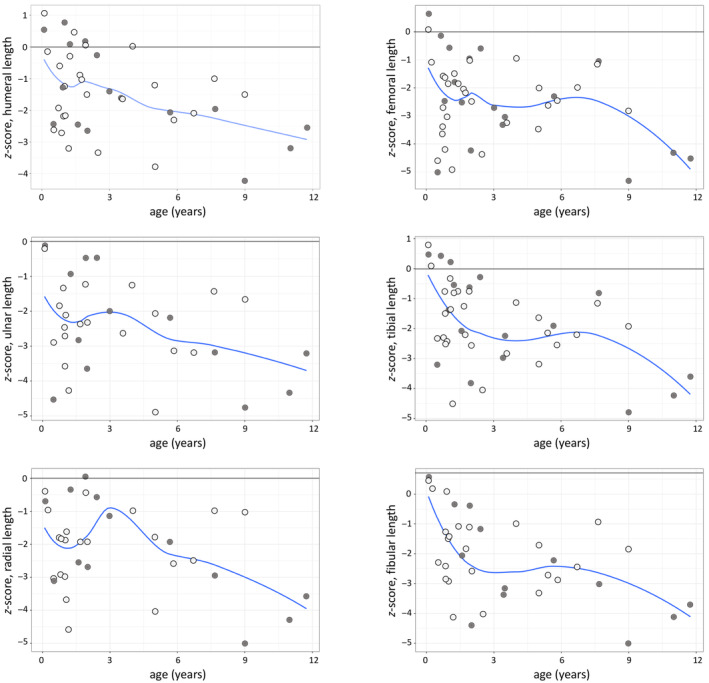
Loess curve fitted to *z*‐score for age data of diaphyseal lengths compared with the Maresh reference means (represented by the “0.00 line”). Gray markers represent male individuals, while white markers represent female individuals.

Figure 2 shows *z*‐scores for humeral, radial, ulnar, femoral, tibial, and fibular diaphyseal length by age of the Certosa children compared with the reference data. The deficit between sample individuals and the reference group increases with age, with 75% of individuals over the age of 5 years qualifying as stunted (≤ −2) in their CZS, compared to 32% of individuals under the age of 2 years. All of the diaphyseal lengths examined produce a stunted mean *z*‐score value (≤ −2), except for the humerus (mean *z*‐score = −1.47) and tibia (mean *z*‐score = −1.81). *Z*‐score values for measurements obtained from the left side were not found to differ significantly from measurements taken from the right side (*p* ≥ 0.05, see Supporting Information S3 for additional results). All diaphyseal length means differ significantly (*p* ≤ 0.01) from the reference data. Four measurements from two individuals were identified as potential outliers (*z*‐score ≤ −5). These measurements were reviewed to ensure their accuracy and were considered to be accurate. The impact of these observations on study findings was explored; results do not change when these individuals are removed from analyses.

**FIGURE 3:**
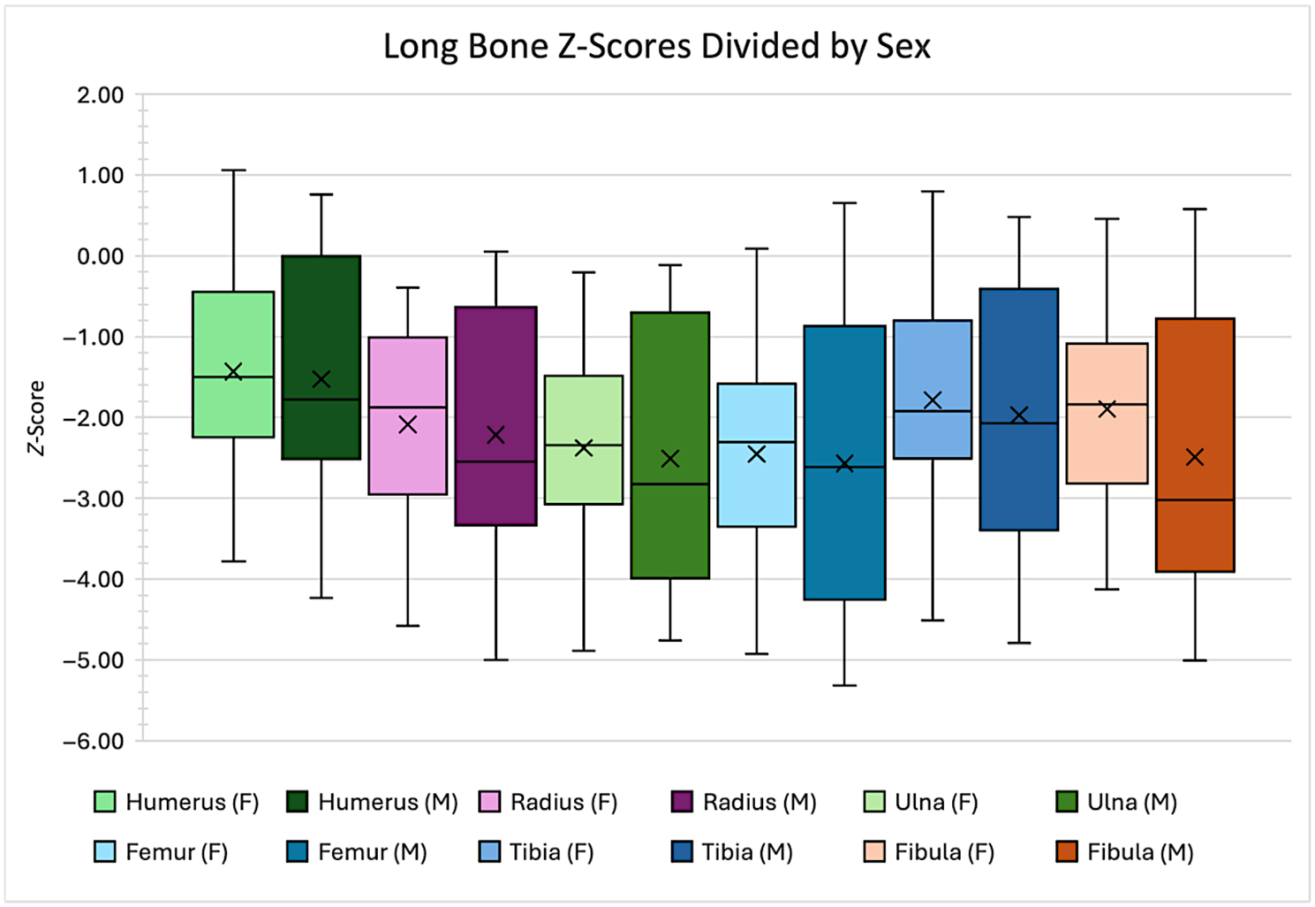
Box plots for each of the six long bones comparing the distribution of female and male *z*‐scores. The *x* represents the mean value, while the median is represented by the line.

When sex groups are considered separately, it is apparent from mean *z*‐score values (Table 5) that diminished growth is more prevalent in males than in females. Although the males in the study sample produce lower mean *z*‐scores for all long bones, the mean *z*‐score differences between the sexes are not statistically significant for any bone (Table 5). The distribution of these differences are demonstrated in Figure 3. This result could be an outcome of the greater number of females in the study sample.

**TABLE 5:** Long bone *z*‐scores summary statistics by sex.

Bone	Females				Males				Independent samples *t*‐test	
	*n*	$\bar{x}$	Median	SD	*n*	$\bar{x}$	Median	SD	*t*	*p*
Humerus	25	−1.43	−1.50	1.233	16	−1.53	−1.78	1.440	0.226	0.822
Radius	21	−2.09	−1.87	1.148	13	−2.21	−2.55	1.593	0.265	0.793
Ulna	20	−2.38	−2.34	1.107	13	−2.51	−2.83	1.627	0.285	0.777
Femur	28	−2.45	−2.31	1.220	18	−2.57	−2.62	1.745	0.272	0.787
Tibia	27	−1.78	−1.92	1.188	17	−1.97	−2.07	1.704	0.231	0.668
Fibula	24	−1.89	−1.84	1.214	13	−2.49	−3.02	1.734	1.227	0.228
CZS	28	−1.97	−1.81	1.118	18	−2.08	−2.26	1.601	0.169	0.866

Abbreviations: *n*, number of individuals; SD, standard deviation for *z*‐score values; $\bar{x}$, mean *z*‐score value.

### 3.3 Upper Versus Lower Limb

**FIGURE 4:**
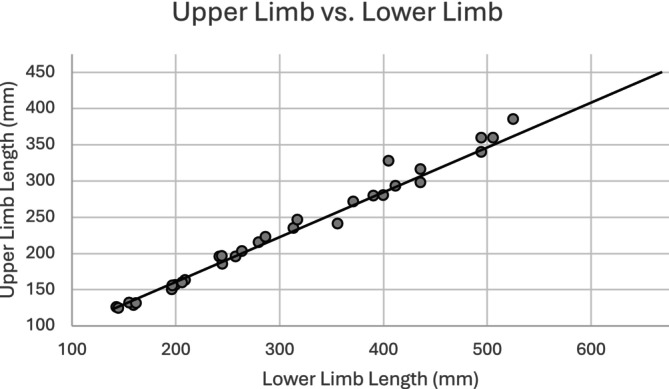
Lower limb length versus upper limb length (sexes pooled). The line represents the profile of the reference sample (Spake and Cardoso 2021).

Analysis of the mean *z*‐scores between the upper and lower limbs indicate differences in their growth. The *z*‐score values (Table 6) indicate that the lower limb is more diminished in growth than the upper limb. Figure 4 shows lower limb length plotted against upper limb length, with a reference line representing the mean values from the reference sample (Spake and Cardoso 2021). If limb proportions in the study sample follow the same growth profile as the reference population, the observations would be located on the reference line. The majority of the observations are located on or slightly above the reference line, indicating that lower limb length in the study sample tends to be slightly shorter relative to upper limb length. A paired samples *t*‐test indicates the difference between the mean *z*‐score of the upper limb and lower limb is statistically significant (*p* ≤ 0.001), with the lower limb having more diminished growth relative to the upper limb.

**TABLE 6:** Upper and lower limb *z*‐scores summary statistics (sexes pooled).

Upper limb				Lower limb				*p*
*n*	$\bar{x}$	Median	SD	*n*	$\bar{x}$	Median	SD	
43	−1.74	−1.62	1.3072	46	−2.23	−2.24	1.3887	< 0.001

Abbreviations: *n*, number of individuals; SD, standard deviation for *z*‐score values; $\bar{x}$, mean *z*‐score value.

### 3.4 Proximal Versus Distal Limb Segments

#### 3.4.1 Upper Limb

**FIGURE 5:**
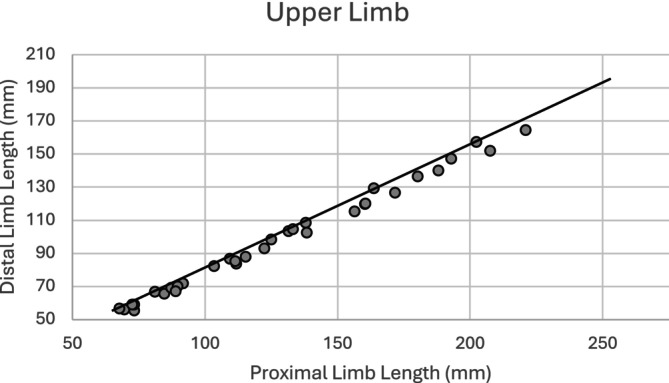
Distal limb segment length (average of ulnar and radial diaphyseal lengths) versus proximal limb segment length of the upper limb (sexes pooled). The line represents the profile of the reference sample (Spake and Cardoso 2021).

Comparisons of the mean *z*‐score values of the proximal and distal limb segments demonstrate differing growth between the two. In the upper limb, the mean *z*‐score of the ulna and radius is significantly lower than that of the humerus (Table 7), indicating more delayed growth in the distal segment of the upper limb relative to its proximal segment. Figure 5 shows the distal segment of the upper limb plotted against the proximal limb segment, with a reference line representing the mean values from the reference sample (Spake and Cardoso 2021). The majority of the observations are located below the reference line, indicating that in the upper limb, proximal limb segment length in the study sample tends to be consistently longer relative to distal limb segment length when compared to the profile from the reference population. The difference between mean *z*‐score values for the proximal and distal segments of the upper limb was found to be significant by a paired samples *t*‐test (*p* ≤ 0.001), with the distal segment demonstrating greater growth faltering than the proximal segment in the upper limb. When the bones of the distal segment are compared separately to the proximal segment, the difference is still found to be significant (ulna, *p* ≤ 0.001; radius, *p* ≤ 0.001). This significance persists when the sexes are evaluated separately (males, *p* ≤ 0.001; females, *p* ≤ 0.001).

**TABLE 7:** Upper limb *z*‐scores summary statistics (sexes pooled).

Distal segment				Proximal segment				*p*
n	$\bar{x}$	Median	*SD*	n	$\bar{x}$	Median	*SD*	
35	−2.26	−2.12	1.2785	41	−1.47	−1.50	1.3022	< 0.001

Abbreviations: *n*, number of individuals; SD, standard deviation for *z*‐score values; $\bar{x}$, mean *z*‐score value.

#### 3.4.2 Lower Limb

**FIGURE 6:**
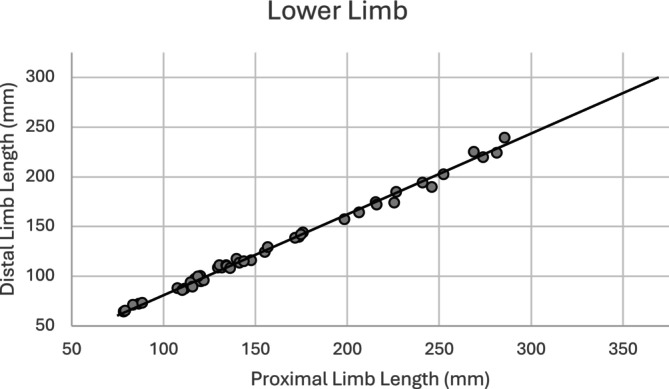
Distal limb segment length (average of tibial and fibular diaphyseal lengths) versus proximal limb segment length of the lower limb (sexes pooled). The line represents the profile of the reference sample (Spake and Cardoso 2021).

Evaluation of the proximal and distal segments in the lower limb reveals a different trend, with mean *z*‐score values of the femur being lower than those of the tibia and fibula (Table 8). This shows that the proximal segment of the lower limb tends to be more delayed in growth relative to the distal segment. Figure 6 shows the distal segment of the lower limb plotted against the proximal limb segment, with a reference line representing the mean values from the reference sample (Spake and Cardoso 2021). The majority of the observations are located on or slightly above the reference line, indicating that in the lower limb, proximal limb segment length in the study sample tends to be shorter relative to distal limb segment length when compared to the profile from the reference population. A paired samples *t*‐test (*p* ≤ 0.001) revealed the proximal segment of the lower limb was significantly more diminished in growth relative to the distal segment. This significance persists when the bones of the distal segment are compared separately to the proximal segment (tibia, *p* ≤ 0.001; fibula, *p* ≤ 0.014). This significance also persists when the sexes are evaluated separately (males, *p* ≤ 0.008; females, *p* ≤ 0.001).

**TABLE 8:** Lower limb *z*‐scores summary statistics (sexes pooled).

Distal segment				Proximal segment				*p*
*n*	$\bar{x}$	Median	SD	*n*	$\bar{x}$	Median	SD	
44	−1.88	−1.95	1.4010	46	−2.50	−2.47	1.4310	< 0.001

Abbreviations: *n*, number of individuals; SD, standard deviation for *z*‐score values; $\bar{x}$, mean *z*‐score value.

Results from the comparative analyses conducted using sex‐specific formulae created from an alternative dataset (Cardoso, Abrantes, and Humphrey 2014) are provided in Table 9, displayed as the mean discrepancies between expected diaphyseal length based on known age and actual length for each bone. The discrepancy of the humerus (mean = −0.79 mm) was significantly different (*p* = 0.001) than the mean discrepancies of the radius (mean = −2.74 mm) and ulna (mean = −3.35 mm). In the lower limb, the discrepancy of the tibia (mean = 1.22 mm) was significantly different (*p* ≤ 0.001) than that of both the femur (mean = −2.58 mm) and fibula (mean = −2.23 mm). These results are consistent with those found using the Denver dataset for *z*‐score calculations.

**TABLE 9:** Discrepancies between predicted length‐for‐age and actual length (mm) for each long bone (sexes pooled) using Cardoso, Abrantes, and Humphrey (2014) formulae.

Humerus			Radius			Ulna		
*n*	$\bar{x}$	SD	*n*	$\bar{x}$	SD	*n*	$\bar{x}$	SD
41	−0.79	9.12	34	−2.74	8.30	33	−3.35	8.36

Femur			Tibia			Fibula		
*n*	$\bar{x}$	SD	*n*	$\bar{x}$	SD	*n*	$\bar{x}$	SD
46	−2.58	14.52	44	1.22	12.32	37	−2.23	11.95

Abbreviations: *n*, number of individuals; SD, standard deviation for diaphyseal length discrepancy (mm); $\bar{x}$, mean diaphyseal length discrepancy (mm).

## 4 Discussion

The children of the Certosa collection demonstrate delayed or diminished linear skeletal growth in comparison to the Denver reference sample, with stunting (≤ −2 SD) in four out of six long bones. It is apparent from study findings that variation exists in the development of the limbs and limb segments in the Certosa children. The lower limb was found to be more delayed in growth relative to the upper limb, and the distal segment of the upper limb exhibited a greater growth deficit than was observed in its proximal segment. These results are consistent with the laws of developmental direction, which predict increased sensitivity to deleterious growth environments in those skeletal elements furthest from the head and trunk. In contrast to the upper limb, the bones of the lower limb display a different pattern of affected growth, where the proximal bone was found to be significantly more delayed in growth in comparison to both bones of the distal segment. This finding does not align with the anticipated study outcomes based on the theorized developmental gradient. The differences in growth delay and deviation from expected findings based on existing literature warrant further consideration in light of the sample context, such as maternal health, documented illness, and nutrition.

Given the impoverished background of the children buried in this section of the cemetery, the finding of diminished growth in this population is not unexpected and aligns with previous research into the collection (Nelson et al. 2020, 2021), which found the vertebral and dental development of these children to be reduced when compared to existing reference standards (Hinck, Hopkins, and Savara 1962; Hinck, Hopkins, and Clark 1965; Hinck, Clark, and Hopkins 1966; Cardoso, Spake, and Liversidge 2016; Cardoso et al. 2019). As growth of dental and spinal tissues is considered to be more resilient to environmental perturbations than bones of the appendicular skeleton (Cardoso 2007; Clark et al. 1986), it is not surprising that the more sensitive diaphyseal long bone lengths of these children also exhibit growth delays.

In examining the prevalence and patterns of diminished growth within the Certosa collection and the possible biocultural factors involved, it is prudent to first discuss issues stemming from the osteological paradox (Wood et al. 1992). As the study sample is composed of children whose biocultural circumstances resulted in their premature death, inherent biases exist when comparatively analyzing their growth against that of living children. It must be acknowledged that the developmental factors which negatively impacted the growth of their skeletal tissues increased the likelihood of them dying, thereby making it more likely that diminished growth will be observed in the study sample than would be seen in a living population. This bias has been dubbed the biological mortality bias (Saunders and Hoppa 1993). Based on this theoretical framework and the increased probability of stunted children dying (de Onis et al. 2019; McDonald et al. 2013; Thurstans et al. 2022), it is likely that those children from the study sample experienced greater diminished growth, as well as differing growth patterns, than what might be observed in individuals who survived past childhood. This is worth noting, as many studies whose results are supportive of the laws of developmental direction were conducted using data from living individuals (Bogin et al. 2002; Buschang 1982; Frisancho 2007; Leung et al. 1996; Takamura et al. 1988; Wadsworth et al. 2002). If the biocultural stress or developmental delay was severe enough to be a contributing factor in the individual's death, it may then be severe enough to impact proximally located skeletal elements. This bias could be behind the unexpected finding of the significantly greater stunting observed in the proximal lower limb segment in the study sample. While this differs from the pattern of diminished growth typically seen in living children experiencing poor biocultural conditions, how it presents in deceased individuals may vary and indicate more severe developmental concerns.

Further to this point, the biological mortality bias may also be present in the age composition of the study sample via selective mortality (Wood et al. 1992). As those individuals in the first years of life experience the greatest mortality, this results in a disproportionately high number of individuals in the youngest age groups, which in turn may impact the prevalence of growth stunting when compared with older individuals. As growth patterns are known to vary with age, where the tempo and timeline for individual skeletal elements do not remain consistent throughout childhood (Harrison 1992; Cunningham, Scheuer, and Black 2016; Sinclair 1989), this may alter the pattern of affected growth and be a contributing factor to the results found in the study sample. Specifically, as long bone growth rates are known to be most rapid within the first years of life (Humphrey 1998), this could result in substantially diminished growth or differing patterns of growth stunting in the youngest individuals of the sample. In particular, the femur undergoes relatively more growth during the first years of life than the other long bones (Cunningham, Scheuer, and Black 2016). This may in turn lead to a higher level of growth stunting observed within the study sample relative to what may be experienced in one with more evenly distributed age groups. In addition to the unequal age distribution of the sample, study findings may also be related to the small number of individuals within the sample, as smaller samples can be impacted from disproportionate effects of extreme values. This limitation should be kept in mind when considering the generalizability of these research outcomes.

The unexpected study findings of greater stunting in the proximal segment of the lower limb relative to its distal segment may be due to issues pertaining to the comparability of the reference dataset and the study sample. Data from the Denver Growth Study has been frequently used in anthropological studies of long bone growth and stature in many populations (Schillaci, Sachdev, and Bhargava 2012). It is one of the only studies of its kind, with no others being identified that match its completeness for longitudinal data of all six long bones from infancy to adulthood. However, concerns have been raised in regard to the use of the Denver sample as an example of typical or normal child development (Cardoso and Magalhães 2011; Spake and Cardoso 2018), with children from the Denver sample being found to be significantly smaller‐for‐age compared to individuals from other reference samples (Fels and Harvard). While the data have been found to conform to modern WHO standards for stature and are therefore suitable for evaluating both stature and femoral growth patterns of diverse skeletal assemblages, issues have been identified in their use for detecting statural stunting. A 2012 study by Schillaci and colleagues found that when using the Denver dataset to assess stature in a sample of children from an Indian population, the analysis resulted in both underestimation and overestimation of stunting prevalence, with patterns varying based on age and sex (Schillaci, Sachdev, and Bhargava 2012). These findings may indicate that individuals in the Denver Growth Study were not experiencing unimpeded growth, which in turn could result in varying limb proportions and impact the results of this study in regards to detecting stunting in the sample population. In order to assess if this is the case, a comparative analysis was conducted using sex‐specific age prediction formulae derived from the Lisbon, Spitalfields, and St. Bride's skeletal collections (Cardoso, Abrantes, and Humphrey 2014) to calculate the expected diaphyseal length based on known age of the study sample. Although this analysis differs in its approach to the data (discrepancy between actual length and length predicted based on age vs. *z*‐score calculation), the pattern of results for proximal versus distal limb segments for the study sample does not differ from that produced from the *z*‐score comparisons with the Maresh sample. The distal segment of the upper limb deviates more from expected length than the proximal segment, while the proximal segment of the lower limb has a greater discrepancy from expected length than either of the distal bones. This indicates that the pattern of differential growth delays seen in the Certosa individuals is not the result of issues with the comparative sample.

A further limitation of this research stems from the cross‐sectional nature of the sample data. As growth itself is longitudinal, studies involving cross‐sectional data are unable to measure growth per se, but rather rely on length‐for‐age measurements as a proxy for growth. Results obtained from cross‐sectional data may not be representative of their population and cannot provide information regarding individual catch‐up growth or growth velocity. Additionally, without the ability to comparatively assess the longitudinal growth of individuals who survived childhood into adulthood with the growth of those children who prematurely died and are included in this study sample, discussion of potential explanations for these research findings and interpretations of early health and development in this population are limited by the paradoxical nature of the data. To mediate the inherent biases present in the interpretation of skeletal data, additional evidence is presented throughout this discussion in the form of historical and archival resources, providing non‐biological data for sample contextualization. These include census data pertaining to infant and child mortality from this time period in northern Italy, as well as statistical records of disease prevalence in the region.

The observed variation in the growth of the limbs and limb segments may be indicative of differing exposure to adverse growth conditions during the developmental period. It has been established that skeletal elements undergo development on unique and varying schedules and tempos (Harrison 1992; Cunningham, Scheuer, and Black 2016; Sinclair 1989), which might then align with varying biocultural factors associated with these developmental periods. As previously mentioned, the differing growth timelines of the various regions of the body is a key component behind the laws of developmental direction. The conceptual framework posits that those elements located toward the head and trunk undergo development earlier than the more distally situated bones (Harrison 1992; Sinclair 1989; Tanner 1962). This in turn leads to a greater amount of development occurring in utero, which is typically considered to provide a buffered growth environment, where the fetus is protected from potentially adverse biocultural factors they may be exposed to postnatally. The increased sensitivity and stunting of distal elements are often attributed to the relatively greater amount of growth they undergo postnatally, where their exposure to negative biocultural factors and more rapid rate of growth may result in heightened levels of diminished growth (Buschang 1982; Frisancho 2007; Martorell 1989; Tanner 1962).

When conducting research using archeological cross‐sectional data, such as the present study, it is not possible to ascertain causal factors behind the observed variation in limb growth, however identifying potential contributors is possible. One such factor is that of maternal health status and the role it may play in skeletal development of the child. In instances of poor maternal health, the benefits of greater in utero development may not be as pronounced and may even result in those bones undergoing more of their growth during the fetal period experiencing negative growth effects. There are many scholarly articles which delve into the effects that maternal biocultural status, such as malnutrition (Black et al. 2008), smoking (Knopik et al. 2012; Lampl, Kuzawa, and Jeanty 2003), illness (Lampl and Jeanty 2004), and psychological stress (Dancause et al. 2012), has on in utero growth and the development of the child. The consequences range from underdeveloped organs and low birth weight to miscarriage. Research has also demonstrated a link between childhood stunting and fetal development, indicating a prenatal origin to growth faltering in children of mothers with poor health (Christian et al. 2013). Although the potential impacts are quite varied, there is substantial evidence for the connection between maternal health and fetal development, demonstrating a clear cause and effect relationship at all stages of the in utero period (McMullen and Mostyn 2009). Clinical research exploring the impact of maternal weight on fetal growth found that the femur experienced a faster rate of growth than the tibia, both in infants born stunted at birth and those who were not stunted at birth (Neufeld et al. 2004). The femur also exhibits comparatively faster rates of prenatal growth when evaluated against the humerus (Mehta and Singh 1972). As those skeletal elements undergoing more rapid rates of growth are considered to be more sensitive to deleterious growth conditions (Bogin and Varela‐Silva 2010), this would result in the femur being more impacted by poor maternal health in utero than both the tibia and the humerus, aligning with the findings of the present study.

As the impoverished nature of the study sample is based on parental SES (Belcastro et al. 2017), it stands to reason that the mothers of the observed individuals would have experienced negative biocultural factors associated with poverty, such as inadequate nutrition, poor sanitation, and disease. When these conditions are present during pregnancy, accompanying health issues and nutrient deficiency can lead to immature fetal development (Pozzi 2002). Archival evidence from this period highlights the poor dietary conditions of the working class in the Bologna region. For example, historical accounts document their diet as being “terrible” and consisting mainly of grains with low nutritional value, primarily corn‐based items such as polenta (Fornasin 1998; Hogan and Kertzer 1987; Livi Bacci 1990). In addition to being comparatively lower in calories than other grains, diets high in corn are also associated with pellagra, a condition stemming from vitamin B3 (niacin) deficiency (Dalla‐Zuanna and Rosina 2011). Records show that the condition was prevalent in northern Italy in the late 19th century (Livi Bacci 1986), with statistical records on population health from this time identifying a link between ill, undernourished parents and frail offspring (Sormanni 1881). This relationship between maternal malnourishment and underweight newborns has also been observed in modern developing countries (WHO Expert Committee on Physical Status 1995).

The effects of this nutrient lacking diet would have been exacerbated by the unsanitary and poor living conditions of Bologna at this time. As a result of Italy's industrial revolution during the 19th century (Kertzer 1987), the city saw a rapid increase in both its population and pollution, with many residents of its surrounding towns moving to the urban center (Hogan and Kertzer 1985). The combination of overcrowding and inadequate sanitation infrastructure led to frequent infections and chronic malnutrition among Bologna's lower socioeconomic classes. In particular, the consumption of unsanitary water from Bologna's well and canal systems has been identified as a cause of many health concerns. Favored by the city's poorer citizens for the free water they provided, these water sources posed an extreme risk to the health of those who used them. Historical documents attribute recurrent and widespread cholera and typhoid epidemics in Bologna to the contaminated water supply (Drusiani et al. 2010; Faggioli 2006; Hogan and Kertzer 1987). These unhygienic conditions and accompanying infectious diseases would have taken a toll on the physical condition of Bologna's impoverished population, with death records for the Certosa collection indicating that approximately 30% of the individuals died as a result of infectious and parasitic diseases, including gastroenteritis (Belcastro et al. 2017).

These factors support the theory that the mothers of the Certosa children experienced poor health, both prior to and during their pregnancies, which in turn may have resulted in poor health and accompanying diminished growth of their offspring. Studies examining the relationship between SES and birth weight have found that neonates from low socioeconomic groups have a greater frequency of low birth weight than those from higher social groups, indicating a link between maternal SES and fetal development (Scalone and Samoggia 2018; Ward 1993). Statistical studies of micro‐data on socioeconomic conditions and high infant mortality rate in northeastern Italy from this time period support the causal effect that poor maternal health would have had on the physical condition of their infants (Dalla‐Zuanna and Rosina 2011; Scalone and Samoggia 2018). Death records from the late 19th century cite “infantile atrophy” as the cause of death in approximately 60% of infants born in the region (Direzione Generale della Statistica 1890). At this time, the condition was diagnosed in instances when an infant was underweight or delayed in their physical development as a result of maternal malnourishment or congenital factors (Manfredini and Pozzi 2004). In cases of poor maternal health, it is expected that fetal development would be impacted from early on in the in utero period. This would explain why even those bones which form relatively early, such as the femur, show diminished growth. Similar results have been found in research into the body proportions of full‐term neonates determined to be small‐for‐gestation, where prolonged periods of intrauterine growth retardation have been linked with growth delays in earlier forming skeletal elements (Brooke, Wood, and Butters 1984).

Further evidence in support of this explanation is found in the differential growth stunting observed between the males and females of the study sample. The Certosa males experienced more diminished growth than the females for all skeletal elements, although not significantly so. These findings are in line with existing literature, which has demonstrated that males are more susceptible to the adverse biocultural conditions found in deleterious growth environments (Stini 1969; Stinson 1985). One possible interpretation of this observation is that individuals within this population did not follow gender‐biased child‐rearing practices. In this situation, the greater sensitivity of males to growth perturbations is not mediated by preferential treatment over their female counterparts, therefore they continue to experience relatively more diminished growth as a result. An examination of data on mortality and disease rates by sex from this time period in Italy does not support this interpretation. Statistical records for northern Italy from the late 19th century indicate that although males did experience a greater risk of neonatal mortality than females (Hogan and Kertzer 1987), the rates reach near equal levels within the first year of life (Manfredini, Breschi, and Fornasin 2017). By the third year of life, risk of death is notably higher in girls (Manfredini, Breschi, and Fornasin 2017), as are rates of disease (Pinnelli and Mancini 1999). These data are suggestive of cultural practices which favored males and put females at a disadvantage. In light of this, an alternative interpretation of the observed sex differentials in growth delays is that the disruption began prior to birth, as it would not be possible to act on gender biases during the fetal period while sex of the child was unknown. This would align with the greater growth deficit observed in the Certosa males, in spite of evidence that they receive preferential treatment and a more favorable postnatal growth environment than the females. It is possible that the growth delays experienced in utero could not be mediated by the advantages they received after birth, which research shows can be difficult to recover from (Chung and Kuzawa 2014; Dewey and Mayers 2011).

This is particularly true for children subject to poor growth environments, such as those present in the study sample. It is very likely that the negative biocultural factors responsible for poor health of the mothers would have impacted the children of the Certosa collection postnatally as well. When an individual experiences long‐term stress, such as chronic malnutrition or illness, throughout their developmental period, it impacts their ability to recover from delayed growth (Dewey and Mayers 2011). Additionally, evidence shows that individuals whose growth is already stunted at the time of stress episodes are unlikely to undergo “catch‐up” growth following cessation of the stressor (Dewey and Mayers 2011), thereby compounding the effects of the growth faltering and falling further off their expected growth curve. In these circumstances, the finding of the femur exhibiting the greatest level of growth stunting could be attributable to it undergoing the relatively greatest amount of growth, in addition to having an extended exposure to negative biocultural factors, during both the in utero and postnatal periods (Cunningham, Scheuer, and Black 2016). Future research into the limb proportions of the adult members of the Certosa collection would offer additional insight into the potential for catch‐up growth in this biocultural environment. Additionally, evaluation of the adult females within the collection has the potential to provide further evidence pertaining to the impact of maternal health status in this population.

While the observation of greater stunting in the proximal segment of the lower limb does not align with the expected research outcomes, this study is not the first to find such a trend. In their analysis of childhood growth throughout periods of religious transitions in Portugal, Gooderham et al. (2019) found the femur to exhibit mean *z*‐scores lower than those of the tibia, while Cardoso (2005) observed femoral growth delay which was equal to or greater than that of the tibia when evaluating the growth of impoverished Portuguese children from the 20th century. Similar results were found by Temple (2014) in their comparison of skeletal growth between temporally distinct groups of foragers in Eastern Siberia. Significantly greater levels of femoral stunting were detected in individuals from the earlier group, who were believed to have experienced increased systemic stress, while no significant difference was found in the tibial lengths of the two groups. An analysis of growth in populations comprised of enslaved African children and impoverished Black communities in 1920s America (Cardoso et al. 2019) revealed that two of the three samples had greater growth stunting in the femur than the tibia. The results found by these and other studies (Holliday and Ruff 2001; Johnston 1962; Pinhasi et al. 2006) indicate that our understanding of growth and varying sensitivity may require further consideration.

Beyond the archeological research cited above, an examination of clinical literature reveals that femoral stunting is not an unusual occurrence in modern health settings, particularly during the prenatal stage. Because of the known association between short femur length and a wide range of adverse health outcomes, the femur is the only long bone required by international guidelines to be routinely measured at fetal ultrasound appointments during the second and third trimester (D'Ambrosio et al. 2019; Li et al. 2022). The measurement is considered to be an early indicator of placental insufficiency (Zalel et al. 2002), intrauterine growth restriction (Bromley, Brown, and Benacerraf 1993; Goetzinger et al. 2012), and low birth weight (Friebe‐Hoffmann et al. 2022; Mailath‐Pokorny et al. 2015), with approximately 20% of fetuses classified as having short femur length being born as small‐for‐gestational age (Mathiesen et al. 2014). Research shows that the postnatal development of children who are born small‐for‐gestational age has the potential to be impacted throughout their life, with approximately 10%–30% of individuals not experiencing catch‐up growth and achieving a stunted terminal stature (Albertsson‐Wikland and Karlberg 1997; Cianfarani, Ladaki, and Geremia 2006; Leger et al. 1997). This suggests that those individuals presenting with reduced femoral lengths early in life may then also be subject to diminished growth overall, particularly in instances where their socioeconomic conditions are not conducive for catch‐up growth, such as those experienced by the children of the Certosa collection. Unfortunately, the identified clinical sources which examine femoral length as an indicator of fetal health do not also provide data on the relative length of the other long bones, particularly the tibia, which limits our ability to interpret archeological evidence of skeletal growth from these elements within the context of clinical research.

## 5 Conclusion

This study explores the differential sensitivity of limb dimensions within the theoretical framework of the laws of developmental direction. By using a documented skeletal collection of known SES, it was possible to assess variability in diminished growth for age in impoverished children. The findings of this research indicate that the children of the Certosa collection demonstrate delayed or diminished skeletal growth in comparison to the reference sample; however, the results also show that the regions of the body are not equally affected by biocultural factors and do not fully align with the theorized developmental gradient. While the lower limb was found to be more delayed in growth relative to the upper limb, and the distal segment of the upper limb exhibited a greater magnitude of diminished growth than was observed in its proximal segment, comparison of the bones in the lower limb found the proximal bone to be significantly more delayed in growth in comparison to both bones of the distal segment. This observation deviates from expected findings based on the laws of developmental direction and brings into question the applicability of assumptions stemming from this theoretical framework. It is apparent from these results that the regions of the body are not equally affected by biocultural factors, with some bones showing markedly more diminished growth than others. It is believed that the varying timeline and tempo of growth observed in these bones are responsible for the differential growth patterns observed in the present study, which in turn may align with biocultural stressors present at different human life history stages. Several areas for future research were identified through this study, including examination of growth patterns in the adults of the Certosa collection, particularly the adult females for evidence of maternal health status in this population, and exploration of differential growth patterns between paired bones of the distal limb segments. The findings of this research indicate that a greater understanding of early life experiences, both at the individual and maternal level, may be gained through the comparative study of diaphyseal growth in both the proximal and distal limbs and their segments.

## Author Contributions

**Jennifer S. Nelson:** conceptualization (lead), data curation (lead), formal analysis (lead), investigation (lead), methodology (lead), writing – original draft (lead), writing – review and editing (lead). **Lesley Harrington:** conceptualization (supporting), funding acquisition (equal), supervision (lead), writing – review and editing (supporting). **Emily Holland:** funding acquisition (equal), supervision (supporting), writing – review and editing (supporting). **Hugo F. V. Cardoso:** conceptualization (supporting), funding acquisition (lead), project administration (lead), supervision (supporting), writing – review and editing (supporting).

## Ethics Statement

This article does not contain any studies involving human participants performed by any of the authors. All research was undertaken following institutional ethics approval.

## Conflicts of Interest

The authors declare no conflicts of interest.

## Supporting information


Supporting Information S1.

Supporting Information S2.

Supporting Information S3.

## Data Availability

The data that support the findings of this study are available from the corresponding author upon reasonable request.
