# Supplementary material for: Exploring the Laws of Developmental Direction Using a Documented Skeletal Collection
Source: Am J Biol Anthropol. 2024 Dec 25;186(1):e25047. doi: 10.1002/ajpa.25047 (PMC11669766; doi:10.1002/ajpa.25047)
Supplement: Supplementary file 1 — Supporting Information S1. [file AJPA-186-e25047-s002.docx]

## Supplementary Material 1

| Summary statistics of diaphyseal length (mm) for each long bone (females) | | | | | | | | | |
| --- | --- | --- | --- | --- | --- | --- | --- | --- | --- |
| Age | Humerus | | | Radius | | | Ulna | | |
| (years) | n | x̄ | *SD* | n | x̄ | *SD* | n | x̄ | *SD* |
| 0 | 2 (1) | 71.59 | 2.64 | 2 (1) | 54.18 | 2.18 | 1 | 59.84 | / |
| 0.5 | 6 (2) | 85.41 | 7.97 | 6 (3) | 63.54 | 4.85 | 6 (3) | 72.04 | 5.49 |
| 1 | 7 (2) | 106.38 | 12.69 | 5 (2) | 73.22 | 10.93 | 4 | 82.99 | 11.76 |
| 2 | 2 (1) | 113.55 | 2.58 | 1 | 83.33 | / | 1 | 92.86 | / |
| 3 | 1 | 138.03 | / | / | / | / | 1 | 108.59 | / |
| 4 | 1 | 156.44 | / | 1 | 109.57 | / | 1 | 121.22 | / |
| 5 | 3 | 153.09 | 12.72 | 3 | 109.29 | 9.11 | 3 | 119.30 | 10.96 |
| 6 | 1 | 171.77 | / | 1 | 122.20 | / | 1 | 131.45 | / |
| 7 | 1 | 193.00 | / | 1 | 141.50 | / | 1 | 153.00 | / |
| 8 | / | / | / | / | / | / | / | / | / |
| 9 | 1 | 202.50 | / | 1 (1) | 152.43 | / | 1 | / | / |
| 10 | / | / | / | / | / | / | / | / | / |
| 11 | / | / | / | / | / | / | / | / | / |
| Age | Femur | | | Tibia | | | Fibula | | |
| (years) | n | x̄ | *SD* | n | x̄ | *SD* | n | x̄ | *SD* |
| 0 | 2 (1) | 82.47 | 4.63 | 2 (1) | 70.04 | 4.96 | 2 | 66.42 | 6.16 |
| 0.5 | 7 | 109.04 | 13.55 | 6 (1) | 91.74 | 9.47 | 6 | 87.61 | 9.46 |
| 1 | 8 (2) | 131.26 | 13.90 | 8 (1) | 107.24 | 13.46 | 6 (3) | 101.50 | 15.19 |
| 2 | 2 | 145.66 | 16.26 | 2 | 116.53 | 1.10 | 2 | 114.18 | 0.33 |
| 3 | 1 | 174.87 | / | 1 | 141.92 | / | / | / | / |
| 4 | 1 | 206.50 | / | 1 | 164.31 | / | 1 | 164.47 | / |
| 5 | 4 | 213.88 | 11.25 | 4 | 171.23 | 8.83 | 4 | 167.67 | 7.75 |
| 6 | 1 | 246.00 | / | 1 | 191.43 | / | 1 | 187.71 | / |
| 7 | 1 | 274.00 | / | 1 | 219.00 | / | 1 | 220.50 | / |
| 8 | / | / | / | / | / | / | / | / | / |
| 9 | 1 | 269.00 | / | 1 | 225.50 | / | 1 | 224.50 | / |
| 10 | / | / | / | / | / | / | / | / | / |
| 11 | / | / | / | / | / | / | / | / | / |

Abbreviations: n, number of individuals (the number of individuals where the right side was substituted for the left is indicated in parentheses); x̄, mean diaphyseal length (mm); SD, standard deviation for diaphyseal length (mm).
