# Supplementary material for: Exploring the Laws of Developmental Direction Using a Documented Skeletal Collection
Source: Am J Biol Anthropol. 2024 Dec 25;186(1):e25047. doi: 10.1002/ajpa.25047 (PMC11669766; doi:10.1002/ajpa.25047)
Supplement: Supplementary file 2 — Supporting Information S2. [file AJPA-186-e25047-s003.docx]

## Supplementary Material 2

| Summary statistics of diaphyseal length (mm) for each long bone (males) | | | | | | | | | |
| --- | --- | --- | --- | --- | --- | --- | --- | --- | --- |
| Age | Humerus | | | Radius | | | Ulna | | |
| (years) | n | x̄ | *SD* | n | x̄ | *SD* | n | x̄ | *SD* |
| 0 | 1 | 67.88 | / | 1 | 52.60 | / | 1 | 61.04 | / |
| 0.5 | 1 (1) | 73.43 | / | 1 (1) | 58.09 | / | 1 | 60.01 | / |
| 1 | 5 (1) | 108.05 | 10.77 | 3 | 84.82 | 8.37 | 3 | 93.39 | 8.22 |
| 2 | 2 | 121.58 | 14.26 | 2 | 90.10 | 10.87 | 2 (1) | 98.72 | 14.84 |
| 3 | 2 | 136.11 | 4.00 | 1 | 101.54 | / | 1 | 108.02 | / |
| 4 | / |  | / | / | / | / | / | / | / |
| 5 | 1 | 163.69 | / | 1 | 123.97 | / | 1 | 134.87 | / |
| 6 | / | / | / | / | / | / | / | / | / |
| 7 | 1 | 188.14 | / | 1 | 134.42 | / | 1 | 145.69 | / |
| 8 | / | / | / | / | / | / | / | / | / |
| 9 | 1 | 180.32 | / | 1 | 129.29 | / | 1 | 143.47 | / |
| 10 | / | / | / | / | / | / | / | / | / |
| 11 | 2 (1) | 214.29 | 9.49 | 2 | 150.59 | 6.94 | 2 | 166.02 | 10.58 |
| Age | Femur | | | Tibia | | | Fibula | | |
| (years) | n | x̄ | *SD* | n | x̄ | *SD* | n | x̄ | *SD* |
| 0 | 1 | 79.15 | / | 1 | 66.24 | / | 1 | 64.25 | / |
| 0.5 | 2 (1) | 100.37 | 23.87 | 2 (1) | 84.29 | 18.48 | / | / | / |
| 1 | 5 (2) | 133.96 | 13.97 | 5 (1) | 112.28 | 10.75 | 3 | 116.14 | 11.19 |
| 2 | 2 | 154.97 | 26.45 | 2 | 127.38 | 22.92 | 2 | 120.05 | 21.72 |
| 3 | 3 | 172.08 | 3.58 | 2 | 144.49 | 5.37 | 2 | 138.20 | 2.23 |
| 4 | / | / | / | / | / | / | / | / | / |
| 5 | 1 (1) | 226.50 | / | 1 (1) | 186.50 | / | 1 | 183.00 | / |
| 6 | / | / | / | / | / | / | / | / | / |
| 7 | 1 | 280.50 | / | 1 (1) | 229.50 | / | 1 | 202.00 | / |
| 8 | / | / | / | / | / | / | / | / | / |
| 9 | 1 | 241.00 | / | 1 | 196.00 | / | 1 | 192.50 | / |
| 10 | / | / | / | / | / | / | / | / | / |
| 11 | 2 | 283.50 | 2.83 | 2 | 233.25 | 12.37 | 2 | 230.00 | 9.19 |

Abbreviations: n, number of individuals (the number of individuals where the right side was substituted for the left is indicated in parentheses); x̄, mean diaphyseal length (mm); SD, standard deviation for diaphyseal length (mm).
