# Supplementary material for: Exploring the Laws of Developmental Direction Using a Documented Skeletal Collection
Source: Am J Biol Anthropol. 2024 Dec 25;186(1):e25047. doi: 10.1002/ajpa.25047 (PMC11669766; doi:10.1002/ajpa.25047)
Supplement: Supplementary file 3 — Supporting Information S3. [file AJPA-186-e25047-s001.docx]

## Supplementary Material 3

| Comparison of mean z-score values for measurements obtained from left vs. right bones (sexes pooled). | | | | | | |
| --- | --- | --- | --- | --- | --- | --- |
|  | Left | | Right | | Two-Samples t-test | |
| Bone | n | x̄ | n | x̄ | *t* | *p* |
| Humerus | 31 | -1.54 | 16 | -1.53 | 2.145 | 0.549 |
| Radius | 26 | -2.08 | 8 | -2.33 | 2.179 | 0.640 |
| Ulna | 28 | -2.51 | 5 | -2.00 | 2.447 | 0.421 |
| Femur | 39 | -2.56 | 7 | -2.17 | 2.206 | 0.585 |
| Tibia | 38 | -1.93 | 6 | -1.367 | 2.447 | 0.424 |
| Fibula | 34 | -2.14 | 3 | -1.70 | 3.182 | 0.555 |

Abbreviations: n, number of individuals; x̄, mean z-score value.
